# Supplementary material for: Long Bone Defect Filling with Bioactive Degradable 3D-Implant: Experimental Study
Source: Biomimetics (Basel). 2023 Mar 28;8(2):138. doi: 10.3390/biomimetics8020138 (PMC10123725; doi:10.3390/biomimetics8020138)
Supplement: Supplementary file 1 [file biomimetics-08-00138-s001.zip › biomimetics-2278828-supplementary.pdf]

Supporting Information of: Long bone defect filling with bioactive degradable 3D-implant: experimental study

Arnold Popkov [1], Natalia Kononovich [1], Gleb Dubinenko [2], Elena Gorbach [1], Alexander Shastov [1], Sergei Tverdokhlebov [2] and Dmitry Popkov [1,*]

[1] Ilizarov National Medical Research Center for Traumatology and Orthopedics, Kurgan 640014, Russia; popkov72@gmail.com (A.P.); n.a.kononovich@mail.ru (N.K.); gorbach.e@mail.ru (E.G.); alshastov@yandex.ru (A.S.)

[2] School of Nuclear Science & Engineering, National Research Tomsk Polytechnic University, Tomsk 634050, Russia; dubinenko.gleb@gmail.com (G.D.); tverd@tpu.ru (S.T.)

* Correspondence: dpopkov@mail.ru

Supplementary Materials and Methods

EDX analysis: Chemical composition of the HA coated implants was studied by energy-dispersive X-ray analysis (EDX) on JEOL JCM-6000 scanning electron microscope (JEOL USA, USA) equipped with energy dispersive X-ray spectrometer JED-2300 Analysis Station Plus. The presence of the Ag in EDX spectra is due to the sputter coating of the surface of samples before SEM and EDX with gold to provide electro conductive layer.

FTIR analysis: The chemical composition of the implants was investigated by attenuated total reflectance (ATR) Fourier-transform infrared spectroscopy (FTIR) on Tensor 27 (Bruker Optik GmbH, Ettlingen, Germany) with a Miracle™ single reflection ATR attachment (PIKE Technologies, Madison, Wisconsin, USA). The measurements were performed with a ZnSe crystal at an incident angle of 45°. All FTIR spectra were recorded in the spectral range of 530 – 4000 cm^{-1} with a resolution of 4 cm^{-1}.

Supporting Figures

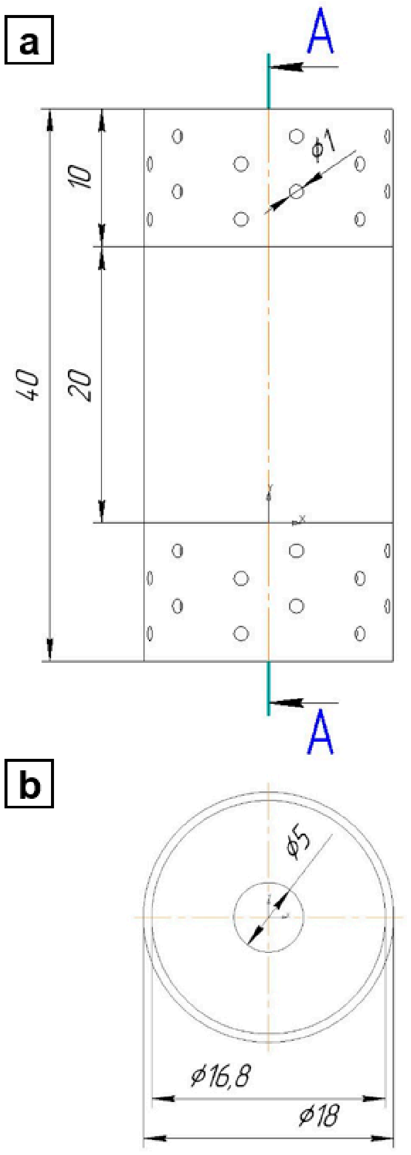

Figure S1. Drawing of the implant: a – front view, b – top view, c – A-A section.

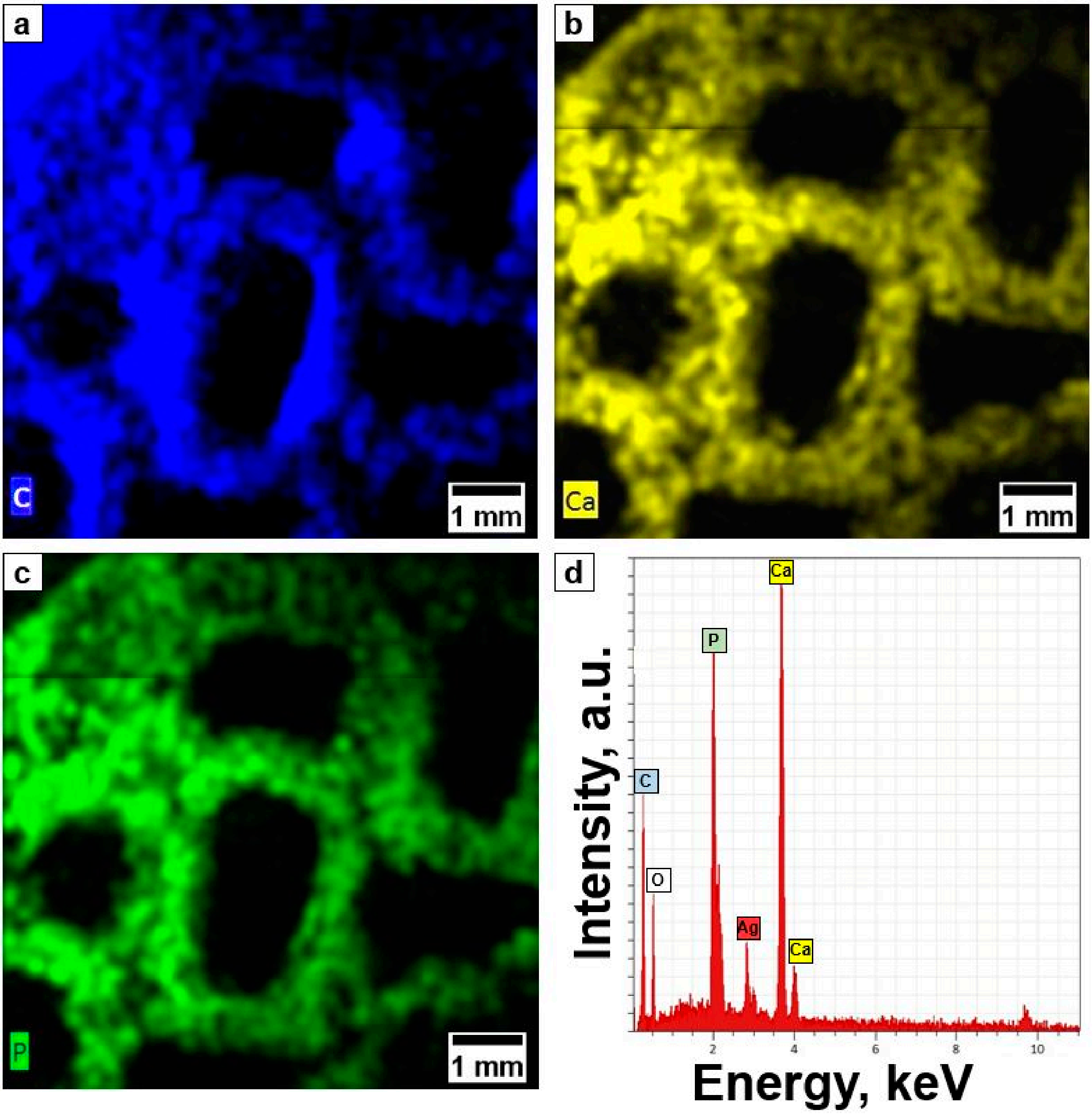

Figure S2. EDX elemental mapping of the HA coated implants: a – carbon (C), b – calcium (Ca), c – phosphorus (P), d – EDX spectra.

FTIR spectra of the scaffolds are shown in Figure S3. The PCL pellets and 3D-printed implant are characterized by the following main bands: 2945 cm^{-1} ($\nu_{as}CH_2$), 2868 cm^{-1} ($\nu_{s}CH_2$), 1724 cm^{-1} (νC=O), 1294 cm^{-1} (ν_sC-O, ν_sC-C), 1240 cm^{-1} (ν_{as}C-O-C) and 1168 cm^{-1} (ν_sC-O-C). In the HA coated implant spectrum, there are also HA-related bands at 1042 cm^{-1} (νPO), 958 cm^{-1} (δPO), 730 cm^{-1} (δPO), and 706 cm^{-1} (δPO) present. There are no significant differences in the shape, width and wavenumber position of PCL bands both in spectrum of the 3D-printed and coated scaffolds. It is important to note that the presence of hydrophilic HA on the surface of the scaffold increases the oxidative reaction of PCL and introduces hydroxyl groups into the polymer backbone. However, there is no evidence for active oxidative degradation of the PCL scaffolds, which is usually indicated by a broadening of the carbonyl peak of the ester groups of polyesters at 1724 cm^{-1}.

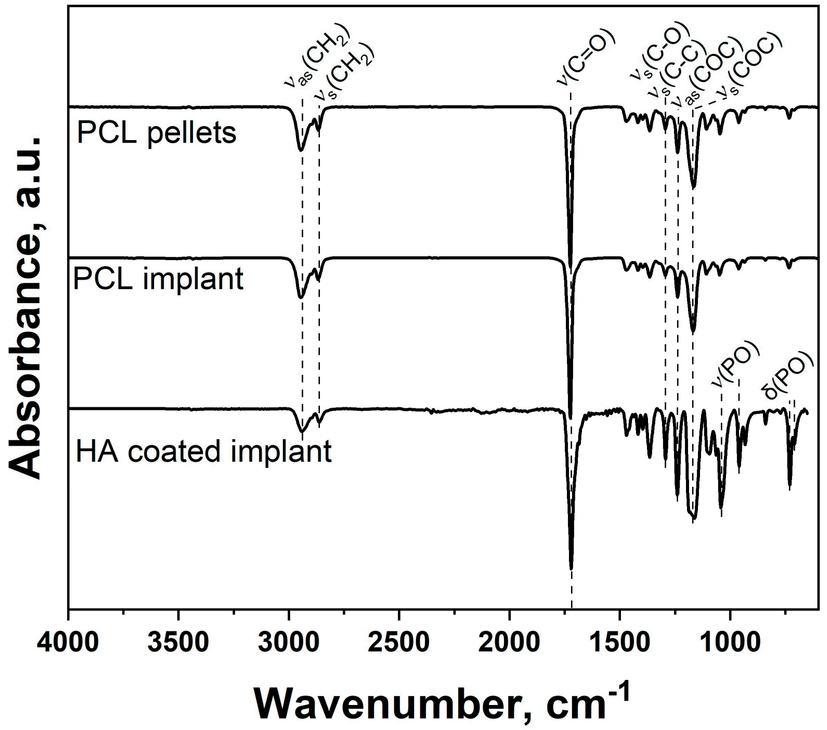

Figure S3. Chemical characterization of the implants by FTIR.
